# A novel technique to maintain a closed reduction of metacarpal fractures

**DOI:** 10.1308/003588412X13373405387096f

**Published:** 2012-05

**Authors:** D Thavarajah, JMW Tibbott, N Hobbs

**Affiliations:** NHS Isle of Wight,UK

## BACKGROUND

**Figure 1:**
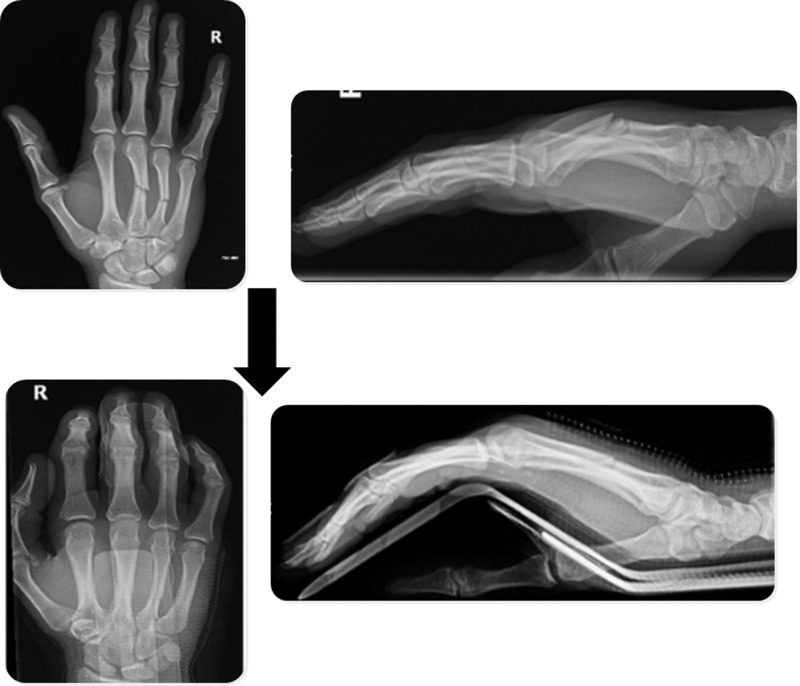
Radiographs demonstrating initial middle and ring displaced metacarpal fractures and radiographs at three-weeks after closed reduction, with splint

Most metacarpal fractures produce a flexion deformity and often minimal shortening. Reduction can frequently be achieved by exerting pressure on the metacarpal head from the palmar aspect, either directly or using the proximal phalanx as a piston. The main issue with the closed technique is maintaining the reduced position in a plaster cast once moulding is complete, thereby commonly necessitating surgical stabilisation. We describe a novel and useful technique that maintains the reduction in the intrinsic plus (Edinburgh) position with the wrist at 20–30º of extension (Fig 1).

## TECHNIQUE

**Figure 2:**
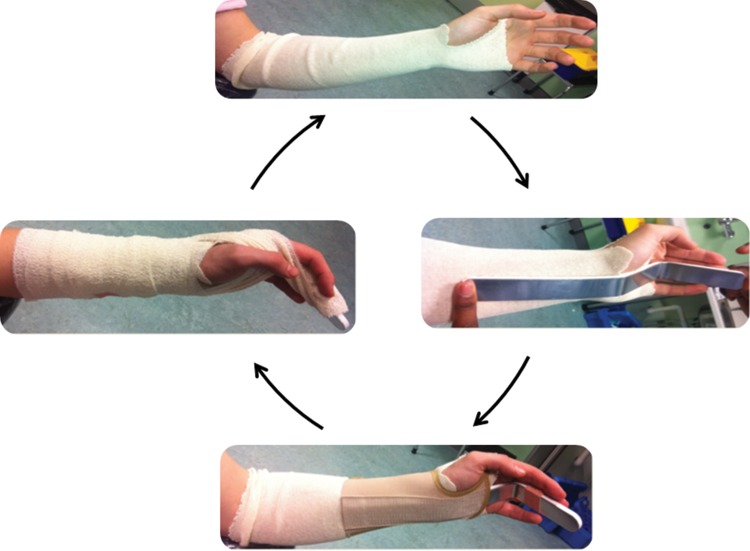
Technique of splinting metacarpal fractures

First, a Tubigrip™ elasticated tubular bandage (Mölnlycke, Dunstable, UK) is applied. An Actimove® Alufoam aluminium finger splint (BSN medical, Hull, UK) is bent into the intrinsic plus position and applied to the volar aspect of the hand and forearm, bridging the fractured metacarpals. The fracture is then reduced as described above. An Actimove® Manus Eco wrist brace is applied snuggly over this. The injured metacarpal fingers are buddied up to the Actimove® Alufoam aluminium finger splint with tape. Finally, a Hospicrepe® Type II cotton crêpe bandage (Paul Hartmann, Heywood, UK) is applied to prevent the patient interfering with the splint (Fig 2).

## DISCUSSION

This technique allows bony union without the need for changing immobilisation materials throughout the fracture healing process, thus enabling continuous stabilisation through conservative management.

